# Development and validation of a nomogram to estimate fatigue probability in hemodialysis patients

**DOI:** 10.1080/0886022X.2024.2396460

**Published:** 2024-09-05

**Authors:** Xinran Qiao, Yan Zhan, Longti Li, Rong Cui

**Affiliations:** a School of Nursing, Hubei University of Medicine, Shiyan, Hubei, China; b Taihe Hospital, Hubei University of Medicine, Shiyan, Hubei, China

**Keywords:** Hemodialysis, fatigue, nomogram, depression

## Abstract

**Objective:**

This study aims to investigate the factors that influence fatigue in hemodialysis (HD) patients and to develop and validate a nomogram to estimate the probability of fatigue in this population.

**Methods:**

This cross-sectional study collected 453 patients who underwent HD at the tertiary hospital in Hubei, China, from April to December 2023. They were randomly divided into a 70% training group (*n* = 316) and a 30% validation group (*n* = 137). In the training set, factors influencing fatigue were screened using multivariate logistic regression analysis, and a nomogram was developed to estimate fatigue probability in HD patients. The discrimination and calibration of the nomogram were validated in both the training and validation sets through the area under the receiver operating characteristic (ROC) curve (AUC) and the Hosmer-Lemeshow (H-L) test.

**Results:**

In the training group, logistic regression showed that age, dialysis vintage, inter-dialysis weight gain, hemoglobin, depression, insomnia, and social support were variables associated with fatigue in HD patients. Based on these factors, a nomogram for assessing fatigue probability in HD patients was developed. The AUC was 0.955 (95% CI: 0.932–0.977) and 0.979 (95% CI: 0.961–0.997) in the training and validation sets. The results from the H-L test indicated a good fit.

**Conclusion:**

The nomogram can evaluate fatigue probability in HD patients and may serve as a convenient clinical tool.

## Introduction

Chronic kidney disease is a significant and growing global burden [1]. When patients progress to end-stage renal disease (ESRD), kidney replacement therapy(KRT) becomes essential for their survival [2]. Hemodialysis (HD) is the most prevalent form of KRT, utilized by around 89% of ESKD patients [3]. It is estimated that the total number of HD patients in China will reach 820,000 by 2025 [4,5]. Unfortunately, individuals undergoing HD may experience symptom burdens that diminish their energy levels and quality of life, with fatigue being a prevalent clinical symptom affecting 61.6–93% of HD patients [6–9].

The international Standardized Outcomes in Nephrology-Hemodialysis (SONG-HD) consensus workshop has identified fatigue as a core outcome for HD patients [10]. It can lead patients to experience persistent weakness, lack of energy, increased risk of cardiovascular disease, psychological burden, reduced quality of life, and limitations in daily activities [11]. Additionally, fatigue is identified as an independent predictive factor for mortality in HD patients [12]. Research has shown that HD patients themselves felt that fatigue needed to be alleviated more than death, cardiovascular disease, mobility, and pain [13]. The causes of fatigue are complex, involving physiological factors like anemia, inflammation, and comorbidities [14]; psychosocial factors including anxiety, depression, sleep quality, and social support [15]; and treatment-related factors, such as dialysis vintage, actual ultrafiltration, and inter-dialysis weight gain [16]. Previous studies have demonstrated that improved anemia can help alleviate fatigue in HD patients [17]. Moreover, aerobic exercise and complementary therapies, such as acupressure, aromatherapy, reflexology, massage, and yoga have also been shown to produce modest improvements in fatigue [18–20].

Most existing tools for identifying fatigue primarily focus on measuring its severity, duration, energy levels, and impact on the body [21]. Still, few studies assess fatigue probability from the perspective of its related factors. Estimating fatigue probability can inform clinical decision-making for individual patients’ situations. A nomogram is a probability assessment model simplifying traditional model formulas into image format [22,23]. This study aimed to evaluate fatigue-associated factors and to develop and validate a nomogram to estimate the probability of fatigue in Chinese HD patients, providing a basis for patients to develop targeted intervention strategies.

## Methods

### Study design and participants

In the cross-sectional study, convenience sampling was used to collect data from patients who received dialysis modality as HD at the blood purification center of a tertiary hospital in Hubei Province between April and December 2023. Inclusion criteria required age ≥ 18 years and HD vintage of more than three months due to renal failure, while exclusion criteria included those with irregular dialysis, severe cognitive impairment, and hearing or speech communication disorders. The study protocol was approved by the Research Ethics Committee of Hubei University of Medicine (2023-RE-023). All subjects signed informed consent.

### Demographics and clinical information

Demographic information included gender, age, education level, marital status, employment, living arrangements, and monthly household income. Clinical data included serum calcium, phosphorus, serum ferritin, serum albumin, serum creatnine, parathyroid hormone, hemoglobin, C-reactive protein, ESRD primary cause, body mass index, dialysis vintage, duration of dialysis, dialysis frequency, inter-dialysis weight gain, dry weight, and actual ultrafiltration. All of the above data were obtained from the Hospital Information System.

### Assessment of fatigue

Our study used the Revised Piper Fatigue Scale (PFS-R) to identify fatigue, published in 1998 by Piper et al. [24]. It is a 22-item self-report scale in which subjects recall fatigue in the past week and describe fatigue along four dimensions: behavioral (6 items), affective meaning (5 items), sensory (5 items), and cognition (6 items). Each item has 11 response categories on a 0–10 metric with verbal descriptors anchoring the endpoints, with higher scores signifying more fatigue. Sum all item scores or sum item scores within a dimension and divide by the total number of items or the number of items within that dimension to obtain a total scale score or a score for that dimension. 0 indicates no fatigue, 1–3 mild, 4–6 moderate, and 7–10 severe. The Chinese version of the PFS-R had a retest reliability of 0.98, and a total scale of Cronbach’s *α* was 0.91, indicating good reliability and validity [25].

### Assessment of candidate variables

#### Anxiety and depression

The Hospital Anxiety and Depression Scale (HADS) is primarily utilized to screen for anxiety and depression among general hospital patients. It consists of 7 items to evaluate anxiety and 7 items to assess depression, with a 21-point total for each category. A score of 8 or more means the presence of anxiety or depression [26]. The Chinese version of HADS demonstrated satisfactory internal consistency and retest reliability, with a Cronbach’s *α* of 0.85 [27].

#### Insomnia

The Athens Insomnia Scale is a self-administered questionnaire used to evaluate sleep quality by evaluating various aspects, such as time taken to fall asleep, frequency of night awakenings, overall sleep quality, and total sleep duration [28]. The scale consists of eight questions, reflecting the individual has slept in the past month. If the total score is 7 or more, insomnia is indicated. The Cronbach’s *α* coefficient for the scale was 0.83 [29].

#### Social support

The Multidimensional Scale of Perceived Social Support (MSPSS) is a tool designed to assess an individual’s self-perception of multidimensional social support [30]. This scale comprises three dimensions: family support, friend support, and other support, totaling 12 entries. Response is a 7-point scale. Scores between 12 and 36 mean low-level support, scores between 37 and 60 mean intermediate level, and scores between 61 and 84 mean high level. The Cronbach’s alpha coefficient for the MSPSS was 0.92 [31].

#### Comorbidity

Charlson comorbidity index (CCI) refers to damage and abnormality of organs or tissues other than the underlying disease, and it evaluates the patient’s comorbidities by points. CCI involves a total of 17 diseases and assigns values according to severity, a score of 0 indicates no comorbidity, 1–2 suggests mild to moderate comorbidities, and a score of 3 or more indicates severe comorbidities [32]. CCI is a valuable tool for predicting disease prognosis [33].

### Data collection

All the data were collected by the researchers with scientific training. The above scores and fatigue questionnaires were completed 30 min before HD. The patient’s demographic information and latest clinical details within three months were extracted from the Hospital Information System on the same day. The researchers were familiar with the investigation and conducted face-to-face data collection with patients who provided signed informed consent before the investigation. Researchers used uniform instructions to guide subjects who did not understand the questions through the questionnaire and collected the questionnaires on the spot.

### Statistical analysis

The research data set was randomly classified into a 70% training cohort and a 30% validation cohort using IBM SPSS Statistics (version 27.0). The training group was utilized for nomogram model development and internal validation, while the validation group was used for external validation. Normally and nonnormally distributed continuous variables are presented as the mean ± standard deviation, median, and interquartile range. Between-group comparisons were conducted using the student’s *t*-test, the Mann–Whitney *U* test; Categorical variables were presented as counts and percentages (%), and between-group comparisons were conducted using the *χ*^2^ test. We selected the candidate variables based on univariate regression analysis with a *p*-value <0.05. To enhance clinical applicability, continuous variables with candidate variables were converted into dichotomous variables based on the optimal cutoff value identified by the Youden index. Youden index can balance sensitivity (true positive rate) and specificity to obtain an optimal threshold [34]. The highest Youden index is 1, which is achieved when sensitivity (true positive rate) and false positive rate (1 − specificity) reach 100 and 0%, respectively [35]. Thus, a threshold with the highest Youden index would be an optimal value that compensates for a high error detection rate (true positive rate) with a low false positive rate.

Subsequently, multivariate logistic regression analysis identified independent factors associated with fatigue in the training set. A nomogram was constructed using R software (Version 4.3.2) to visually represent the Logistic regression model. The model’s performance evaluation was conducted based on the training and validation groups, assessing its calibration and differentiation abilities. Internal test of the nomogram model by Bootstrap repetitive sampling 1000 times. Calibration ability was evaluated by the Hosmer-Lemeshow (H-L) test, and *p* > 0.05 indicated better model consistency. The discriminatory ability was determined by the area under the ROC curve (AUC), with AUC > 0.7 suggesting effective discrimination between fatigued and non-fatigued patients. The closer the AUC was to 1, the more accurate the model.

## Results

### Baseline characteristics

A total of 453 samples were included in our study, 316 in the training set and 137 in the validation set. The incidence of fatigue was observed to be 72.2 and 70.8% in the training and validation groups, respectively. The baseline characteristics of the two sets are depicted in Table 1.

**Table 1.** Baseline characteristics of the training and validation group.

Variable	Training group			Validation group		
	Non-fatigue (*n* = 88)	Fatigue (*n* = 228)	*p*	Non-fatigue (*n* = 31)	Fatigue (*n* = 106)	*p*
Gender			0.811			
Male, *n* (%)	58 (65.9%)	147 (64.5%)		17 (54.8%)	53 (50.0%)	0.635
Age (years)	53.32 ± 15.00	58.96 ± 12.45	0.002	51.71 ± 12.88	58.36 ± 12.60	0.014
Education level			<0.001			0.005
College degree and above, *n* (%)	26 (29.5%)	23 (10.1%)		11 (35.5%)	14 (13.2%)	
Others, *n* (%)	62 (70.5%)	205 (89.9%)		20 (64.5%)	92 (86.8%)	
Marital status			0.361			0.092
Married, *n* (%)	71 (80.7%)	173 (75.9%)		20 (64.5%)	84 (79.2%)	
Employment			<0.001			0.542
Employed, *n* (%)	30 (34.1%)	26 (11.4%)		9 (29.0%)	37 (34.9%)	
Monthly household income			0.002			0.002
≥5000 yuan, *n* (%)	28 (31.8%)	37 (16.2%)		11 (35.5%)	13 (12.3%)	
Living arrangements			<0.001			0.005
Lives alone, *n* (%)	26 (29.5%)	26 (11.4%)		7 (22.6%)	6 (5.7%)	
ESRD primary cause			0.801			0.036
Diabetic nephropathy, *n* (%)	24 (27.3%)	59 (25.9%)		4 (12.9%)	34 (32.1%)	
Others, *n* (%)	64 (72.7%)	169 (74.1%)		27 (87.1%)	72 (67.9%)	
Dialysis vintage (months)	19.00 (8.25, 43.50)	35.50 (18.00, 64.75)	<0.001	21 (10.00, 69.00)	25 (15.00, 71.50)	0.127
Duration of dialysis			0.822			.099
4 h, *n* (%)	85 (96.6%)	219 (96.1%)		26 (83.9%)	99 (93.4%)	
Dialysis frequency			0.508			0.817
5 times/2 weeks, *n* (%)	50 (56.8%)	115 (50.4%)		26 (83.9%)	87 (82.1%)	
Others, *n* (%)	38 (43.2%)	113 (49.6%)		5 (16.1%)	19 (17.9%)	
Body mass index (kg/m^2^)	21.24 ± 3.13	21.90 ± 3.09	0.091	20.40 ± 3.40	21.62 ± 3.61	0.099
Dry weight (kg)	60.19 ± 11.73	62.14 ± 11.51	0.018	55.39 ± 10.53	59.62 ± 12.76	0.095
Inter-dialysis weight gain (%)	4.33 ± 2.30	4.94 ± 2.54	0.049	3.98 ± 1.65	5.35 ± 2.27	0.002
Actual ultrafiltration (ml)	2305.53 ± 1236.45	2666.37 ± 1102.22	0.018	2441.55 ± 1090.54	2840.24 ± 969.96	0.052
Serum calcium (mmol/L)	2.13 ± 0.20	2.09 ± 0.22	0.126	2.16 ± 0.32	2.12 ± 0.23	0.332
Phosphorus (mmol/L)	1.72 (1.30, 2.30)	1.71 (1.33, 2.26)	0.832	1.41 (1.16, 2.02)	1.54 (1.14, 2.06)	0.702
Parathyroid hormone (pg/mL)	264.33 (90.15, 540.75)	360.20 (158.05, 604.10)	0.047	109.00 (54.20, 376.00)	352.00 (184.25, 536.03)	<0.001
Serum ferritin (μg/L)	102.33 (61.52, 172.95)	103.05 (47.47, 267.03)	0.431	139.20 (67.60, 192.80)	136.70 (72.10, 355.10)	0.399
Serum albumin (g/L)	40.52 ± 5.55	39.61 ± 5.84	0.206	41.56 ± 4.27	36.91 ± 7.19	<0.001
Serum creatinine (μmol/L)	745.13 ± 291.76	794.58 ± 302.15	0.189	599.52 ± 361.88	687.61 ± 328.14	0.201
Hemoglobin (g/L)	118.63 ± 19.14	105.08 ± 18.43	<0.001	121.13 ± 26.49	106.23 ± 18.70	<0.001
Creactive protein (mg/L)	1.81 (0.63, 3.60)	14.29 (5.88, 28.50)	<0.001	1.96 (0.73, 4.72)	16.63 (9.30, 22.57)	<0.001
Comorbidity index (score)	1.83 ± 1.56	2.18 ± 1.34	0.051	1.61 ± 1.09	2.06 ± 1.25	0.076
Anxiety, *n* (%)	7 (8.0%)	58 (25.4%)	<0.001	2 (6.5%)	24 (22.6%)	0.043
Depression, *n* (%)	8 (9.1%)	160 (70.2%)	<0.001	3 (9.7%)	71 (67.0%)	<0.001
Insomnia, *n* (%)	5 (5.7%)	121 (53.1%)	<0.001	6 (19.4%)	63 (59.4%)	<0.001
Social support			<0.001			<0.001
High, *n* (%)	74 (84.1%)	65 (28.5%)		27 (87.1%)	43 (40.6%)	
Low or medium, *n* (%)	14 (15.9%)	163 (71.5%)		4 (12.9%)	63 (59.4%)	

*Notes:* Values for continuous variables are given as the means ± standard deviations, medians and interquartile ranges. Categorical variables are expressed as numbers (%).

### Univariate and multivariate logistic regression analysis

Univariate analysis of factors revealed that 16 variables, including age, education level, employment, monthly household income, living arrangements, dialysis vintage, dry weight, inter-dialysis weight gain, actual ultrafiltration, parathyroid hormone, hemoglobin, C-reactive protein, anxiety, depression, insomnia, and social support, were associated with fatigue in HD patients (Table 2).

**Table 2.** Univariate analysis for training group.

Variables	OR (95% CI)	*p*
Age (years)	1.032 (1.013, 1.052)	<0.001
Education level (college degree and above)	0.268 (0.143, 0.502)	<0.001
Employment (employed)	0.249 (0.136, 0.454)	<0.001
Living arrangements (lives alone)	0.307 (0.166, 0.567)	<0.001
Monthly household income (≥5000 yuan)	0.415 (0.235, 0.734)	0.003
Dialysis vintage (months)	1.007 (1.000, 1.014)	0.043
Dry weight (kg)	1.015 (0.993, 1.038)	0.180
Inter-dialysis weight gain (%)	1.117 (1.001, 1.245)	0.047
Actual ultrafiltration (ml)	1.000 (1.000, 1.000)	0.013
Parathyroid hormone (pg/mL)	1.000 (1.000, 1.001)	0.790
Hemoglobin (g/L)	0.961 (0.947, 0.976)	<0.001
C-reactive protein (mg/L)	1.202 (1.133, 1.276)	<0.001
Anxiety	3.948 (1.725, 9.033)	0.001
Insomnia	18.772 (7.338, 48.021)	<0.001
Depression	23.529 (10.784, 51.339)	<0.001
Social support	13.255 (6.993, 21.125)	<0.001

OR: odds ratio; CI: confidence interval.

The candidate variables related to fatigue, identified through univariate analysis, were further analyzed using multivariable regression. Ultimately, seven variables related to fatigue in HD patients were determined. These factors, ranked by their contribution, were depression (odds ratio (OR): 18.960, 95% confidence interval (CI): 6.683–53.792), insomnia (OR: 8.591, 95%CI: 2.680–27.538), age (OR: 8.416, 95%CI: 3.233–21.980), dialysis vintage (OR: 7.936, 95%CI: 2.925–21.528), social support (OR: 5.000, 95%CI: 2.030–12.314), inter-dialysis weight gain (OR: 3.494, 95%CI: 1.406–8.680), and hemoglobin (OR: 3.172, 95%CI: 1.340–7.512) (Table 3).

**Table 3.** Multivariate logistic regression analysis of associated factors for fatigue.

Variables	OR (95% CI)	*p*
Age (>50years)	8.416 (3.233, 21.980)	<0.001
Dialysis vintage (>1year)	7.936 (2.925, 21.528)	<0.001
Inter-dialysis weight gain (>3.4%)	3.494 (1.406, 8.680)	0.007
Hemoglobin (>110 g/L)	3.172 (1.340, 7.512)	0.009
Insomnia	8.591 (2.680, 27.538)	<0.001
Depression	18.960 (6.683, 53.792)	<0.001
Social support	5.000 (2.030, 12.314)	<0.001

OR: odds ratio; CI: confidence interval.

### Development of the nomogram in the training cohort

**Figure 1.**
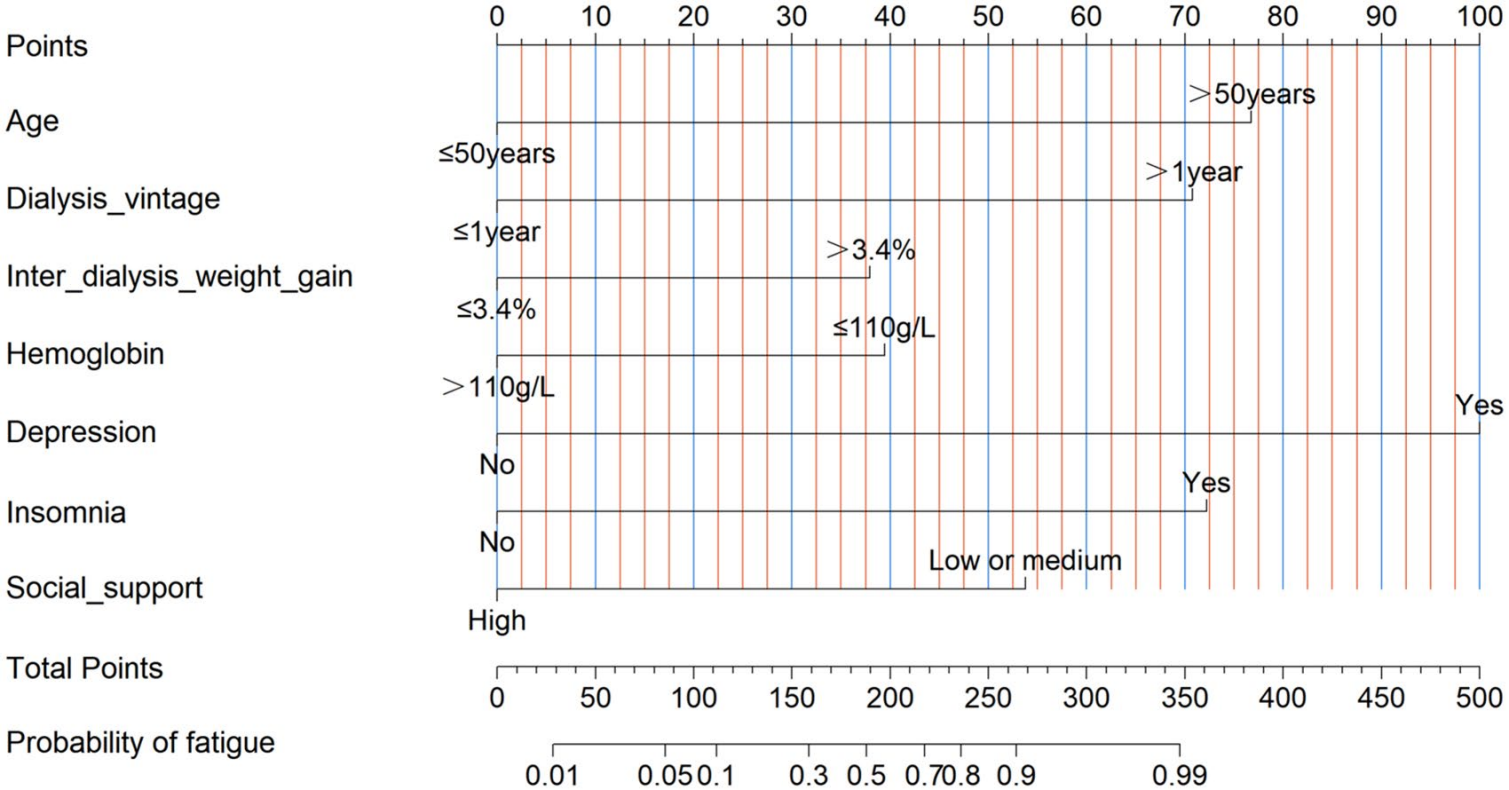
A nomogram of estimate fatigue probability in hemodialysis patients. *Notes:* Placing each variable by its value on the nomogram, a line is drawn upwards at a 90° angle to determine the number of points for that particular variable. The points for each variable are then summed, and this total corresponds to a specific value on the total points axis. Subsequently, a line is drawn downward at a 90° angle to ascertain the fatigue probability. Age (>50 years) = 77 points, dialysis vintage (>1 year) = 71 points, inter-dialysis weight gain (>3.4%) = 38 points, hemoglobin (≤110 g/L) = 39 points, depression (yes) = 100 points, insomnia (yes) = 72 points, social support (low or medium) = 53 points.

Based on these seven associated factors, a nomogram of fatigue in HD patients was developed (Figure 1). Placing each variable by its value on the nomogram, a line is drawn upwards at a 90° angle to determine the number of points for that particular variable. The points for each variable are then summed, and this total corresponds to a specific value on the total points axis. Subsequently, a line is drawn downward at a 90° angle to ascertain the fatigue probability. For example, a 46-year-old patient with 10 months of dialysis, an inter-dialysis weight gain of 2.4%, a hemoglobin level of 105 g/L, depression, insomnia, and a high level of social support would have scores of 0, 0, 0, 39, 100, 72, and 0, respectively. The total score is 211, resulting in a corresponding fatigue probability of about 0.65.

### Discrimination and calibration

**Figure 2.**
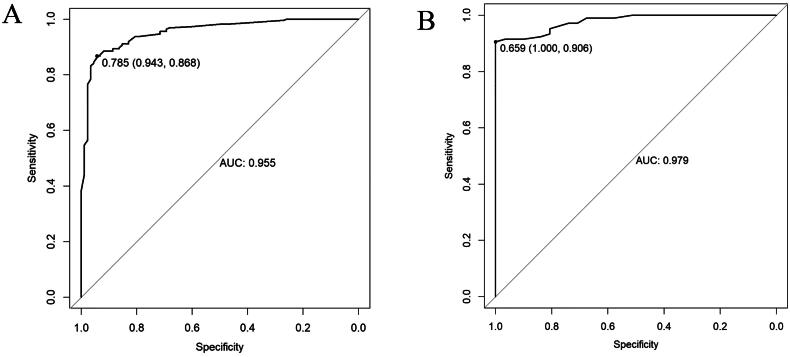
ROC curves of nomogram in the training set (A) and validation set (B).

**Figure 3.**
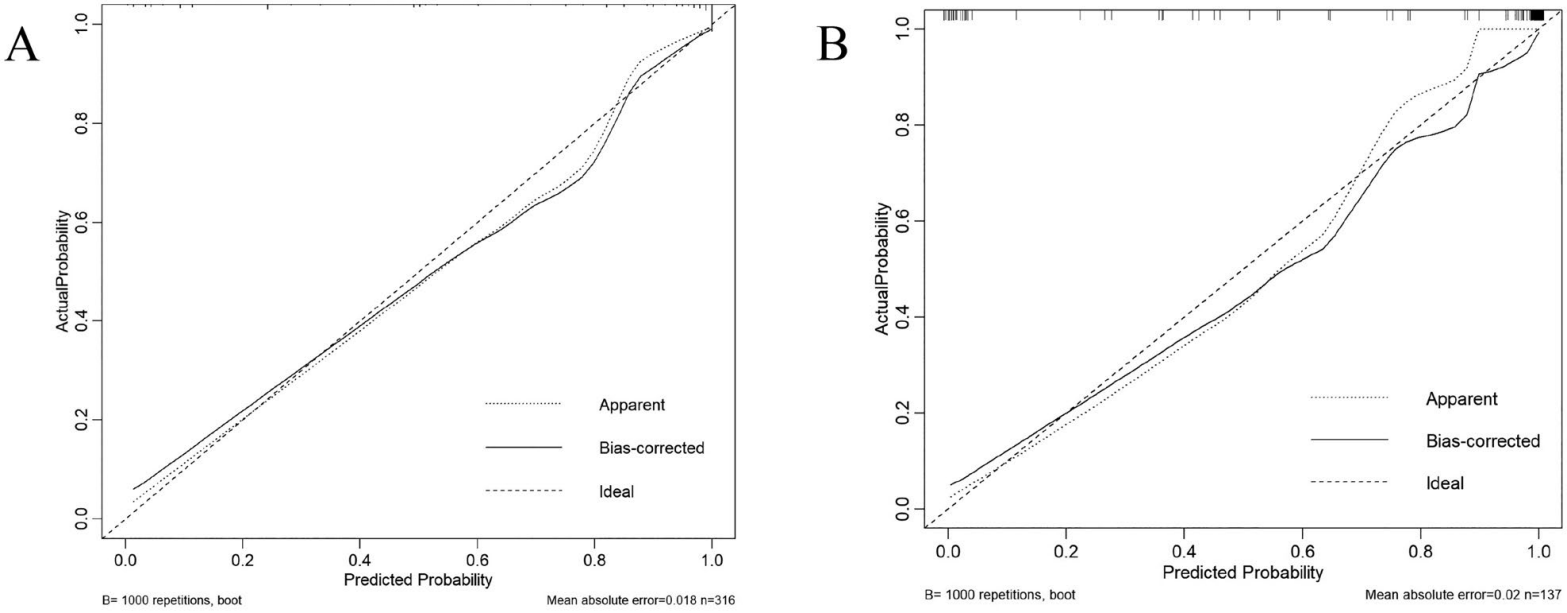
The calibration curves for the Hosmer-Lemeshow model calibration in the training (A) and validation (B) sets.

ROC analysis was conducted based on the probabilities obtained from logistic regression analysis. The nomogram had an AUC of 0.955 (95% CI: 0.932–0.977) for the training data and an AUC of 0.979 (95% CI: 0.961–0.997) for the validation data. In the training group, the optimal cutoff value was 0.785, with a specificity of 0.943, and a sensitivity of 0.868. In the validation group, the optimal cutoff value was 0.659, with a specificity of 1.000 and a sensitivity of 0.906 (Figure 2). The results of the H-L test showed *χ*^2^ = 3.469, *p* = 0.902 for the training set, *χ*^2^ = 8.308, *p* = 0.306 for the validation set. The calibration curves of both sets closely matched the ideal curves, indicating a well-fitted nomogram model (Figure 3).

## Discussion

In the present study, we developed a nomogram plot to assess the fatigue probability in HD patients. The model included seven variables: age, dialysis vintage, inter-dialysis weight gain, hemoglobin, depression, insomnia, and social support. Calibration ability was assessed using the AUC and H-L test, showing good discrimination and accuracy in the nomogram. This nomogram can assist medical personnel in evaluating the probability of fatigue among HD patients, thereby providing a foundation for the early personalization of interventions.

Depression was an independently associated factor of fatigue among HD patients in our study. The prevalence of depression was higher in these patients [36]. Depression can result in poor mental status, endocrine disruption, and decreased energy and stamina in HD patients, leading to physical or mental fatigue [37]. Depression and fatigue also interact with each other, with HD patients experiencing higher levels of fatigue also being at greater risk of depression [38]. Our research revealed a significant positive relation between insomnia and fatigue in these patients, with those experiencing insomnia being 8.6 times more likely to suffer from fatigue compared to others. Previous studies have also highlighted that fatigue levels are elevated in HD patients with insomnia [39]. Dialysis patients can suffer from fatigue, itchy skin, body aches, and other undesirable symptoms that affect the quality of their sleep, similarly, poor sleep can affect the emotional and physical energy of HD patients, causing them to suffer from fatigue [39,40]. A study at the Stanford Sleep Medicine Center demonstrated that insomnia and daytime sleepiness are strong predictors of fatigue, and managing insomnia can effectively reduce fatigue [41]. Therefore, caregivers need to focus on assessing and improving patient’s sleep quality while combating fatigue.

The study revealed a positive correlation between age and fatigue incidence in HD patients, consistent with previous research [42]. Older patients, who are more likely to have comorbidities, malnutrition, and cardiovascular issues compared to younger patients, exhibit lower tolerance to HD treatment, leading to a higher prevalence of fatigue [43]. Additionally, the incidence of fatigue was found to increase in patients with a dialysis vintage exceeding one year, consistent with Zhu’s results [44]. However, a study in Pakistan reported higher fatigue levels in patients with a dialysis vintage exceeding four years [45]. Longer dialysis vintage may predispose patients to inflammation, complications, malnutrition, and other factors contributing to fatigue [46]. Furthermore, hemoglobin levels were inversely related to fatigue in this study, with patients having hemoglobin ≤ 110 g/L showing a 3.2-fold higher incidence of fatigue compared to others. However, the specific hemoglobin value remains debated, as a prior study suggested lower fatigue scores in patients with hemoglobin more than 10 g/dL [47]. Anemia is a prevalent complication in HD patients, with anemic individuals experiencing fatigue symptoms like physical weakness and poor concentration [48]. Therefore, efforts should be made to actively address renal anemia in HD patients through regular laboratory monitoring and timely interventions.

Another significant discovery was the association between fatigue and inter-dialysis weight gain in HD patients, mirroring Gobbi’s research [16]. Gobbi’s study in Italy indicated that fatigue prevalence would rise in patients with inter-dialysis weight gain surpassing 2% of dry body weight. In contrast, our study set the cutoff point at 3.4%. This discrepancy may be attributed to variations in sample size, geographical locations, and cultural influences. Patients often consume more calories and fluids when experiencing fatigue in an attempt to combat their body’s exhaustion through eating. Nevertheless, this can lead to a vicious cycle where increased fluid intake contributes to heightened ultrafiltration. The research also indicates that high-level social support serves as a protective factor against fatigue in HD patients, aligning with findings from a previous study [49]. Social support plays a crucial role in enhancing a patient’s long-term prognosis. Robust social support systems, including family, marital support, and higher income, not only reduce the likelihood of fatigue but also enhance the overall quality of life and mental health of patients [50]. Engaging in social activities like sports and exercise can provide opportunities for patients to reintegrate into society, boost their confidence, and alleviate the psychological burden of feeling isolated [51].

## Conclusion

Our study incorporated seven independent associated factors that influence fatigue in HD patients: age, dialysis vintage, inter-dialysis weight gain, hemoglobin, depression, insomnia, and social support. The resulting HD patient fatigue probability nomogram demonstrates outstanding identification capabilities. This tool can be used by clinical medical personnel to evaluate the probability of fatigue in HD patients.

## Limitation

There are some limitations to our findings. This study has a small sample size and is single-center. Future research should consider prospective, multicenter, and large-sample studies. Furthermore, this study was cross-sectional, future studies could benefit from longitudinal designs to track the trajectory of fatigue in HD patients. The study does not distinguish post-dialysis fatigue from a more cumulative experience of fatigue. Moreover, the fatigue score does not distinguish between individuals who may describe their fatigue in different ways (e.g., sleepiness, muscle weakness, difficulty concentrating, etc.). Patients in this study may have had dialysis intervals of 2 or 3 days, which may affect specific inter-dialysis weight gain, and we look forward to exploring these issues further in future studies.

## Supplementary Material

Supplemental Material
