# Supplementary material for: Development and validation of a nomogram to estimate fatigue probability in hemodialysis patients
Source: Ren Fail. 2024 Sep 5;46(2):2396460. doi: 10.1080/0886022X.2024.2396460 (PMC11382713; doi:10.1080/0886022X.2024.2396460)
Supplement: Supplemental Material [file IRNF_A_2396460_SM6442.docx]

Supplementary table Optimal cutoff values for statistically significant variables

| Variable | cutoff values | Sensitivity | Specificity | Youden index |
| --- | --- | --- | --- | --- |
| Age (years) | 49.5 | 0.851 | 0.523 | 0.374 |
| Dialysis vintage (months) | 11.5 | 0.899 | 0.386 | 0.285 |
| Inter-dialysis weight gain (%) | 3.4 | 0.763 | 0.386 | 0.149 |
| Actual ultrafiltration (ml) | 1850 | 0.794 | 0.386 | 0.180 |
| Parathyroid hormone (pg/ml) | 98.3 | 0.895 | 0.273 | 0.168 |
| C-reactive protein (mg/L) | 4.46 | 0.816 | 0.841 | 0.657 |

Notes: Using the occurrence of fatigue as a state variable, statistically significant continuous variables that could not be easily categorized were analyzed using ROC curves, and the optimal cutoff value was determined based on the maximum Youden index, which was converted to a dichotomous variable.
